# TLR7-mediated skin inflammation remotely triggers chemokine expression and leukocyte accumulation in the brain

**DOI:** 10.1186/s12974-016-0562-2

**Published:** 2016-05-09

**Authors:** Alison McColl, Carolyn A. Thomson, Louis Nerurkar, Gerard J. Graham, Jonathan Cavanagh

**Affiliations:** Institute of Infection, Immunity & Inflammation, College of Medical & Veterinary Life Sciences, University of Glasgow, 120 University Place, Glasgow, G12 8TA UK; Institute of Health & Wellbeing, College of Medical & Veterinary Life Sciences, University of Glasgow, Queen Elizabeth University Hospital, Glasgow, G51 4TF UK

**Keywords:** Peripheral inflammation, Chemokines, Neuroinflammation, Leukocyte infiltration, Burrowing behaviour, Neurogenesis

## Abstract

**Background:**

The relationship between the brain and the immune system has become increasingly topical as, although it is immune-specialised, the CNS is not free from the influences of the immune system. Recent data indicate that peripheral immune stimulation can significantly affect the CNS. But the mechanisms underpinning this relationship remain unclear. The standard approach to understanding this relationship has relied on systemic immune activation using bacterial components, finding that immune mediators, such as cytokines, can have a significant effect on brain function and behaviour. More rarely have studies used disease models that are representative of human disorders.

**Methods:**

Here we use a well-characterised animal model of psoriasis-like skin inflammation—imiquimod—to investigate the effects of tissue-specific peripheral inflammation on the brain. We used full genome array, flow cytometry analysis of immune cell infiltration, doublecortin staining for neural precursor cells and a behavioural read-out exploiting natural burrowing behaviour.

**Results:**

We found that a number of genes are upregulated in the brain following treatment, amongst which is a subset of inflammatory chemokines (CCL3, CCL5, CCL9, CXCL10, CXCL13, CXCL16 and CCR5). Strikingly, this model induced the infiltration of a number of immune cell subsets into the brain parenchyma, including T cells, NK cells and myeloid cells, along with a reduction in neurogenesis and a suppression of burrowing activity.

**Conclusions:**

These findings demonstrate that cutaneous, peripheral immune stimulation is associated with significant leukocyte infiltration into the brain and suggest that chemokines may be amongst the key mediators driving this response.

**Electronic supplementary material:**

The online version of this article (doi:10.1186/s12974-016-0562-2) contains supplementary material, which is available to authorized users.

## Background

Neuropsychiatric disorders, including depression, anxiety and schizophrenia, are a significant and growing burden to society [1] and are also commonly associated with chronic inflammatory disorders [2]. However, we know very little about the mechanisms underpinning this relationship.

Increasingly compelling data have highlighted possible roles for a number of immune mediators, including inflammatory cytokines, in the pathogenesis of neuropsychiatric disorders. Examples include (a) treatment of hepatitis C with IFN-α results in the onset of major depressive disorder (MDD) in around 50 % of patients and is the most common reason for ceasing treatment [3–5], (b) the treatment of psoriasis patients with the anti-TNF drug etanercept improved the clinical symptoms of depression independently of an improvement in psoriatic disease score in patients [6, 7] and (c) patients suffering from long-term MDD or schizophrenia have an elevated inflammatory profile [8, 9]. In essence these pathology-, or therapy-, associated depressive episodes represent exaggerated presentations of the so-called ‘sickness behaviour’, which is a recognised and normal response typically associated with microbial infection. Nonetheless, despite these correlations, it remains unclear how inflammation in the periphery relates to neuropsychiatric phenotypes.

One family of cytokines that might play a key role in this communication pathway is the chemotactic cytokine, or chemokine, family. The classical role of chemokines is in mediating cell trafficking and inflammatory chemokines are important for cell migration during immune and inflammatory responses [10]. Many chemokines, such as CXCL12, are expressed in the brain in steady-state and play an important role in development and homeostasis [11]. However, some inflammatory chemokines are also expressed in the brain and are upregulated during infection [12, 13]. We hypothesised that, in systemic inflammatory disease contexts, brain-expressed chemokines would drive immune cell infiltration into the brain, leading to the activation of local cells resulting in co-morbid psychiatric disorders [14–16].

To test this hypothesis, we used a well-characterised mouse model of skin inflammation. The topical application of Aldara™, a commercially available drug which contains the Toll-like receptor (TLR) 7/8 ligand imiquimod (IMQ), to the dorsal skin of mice, causes a psoriasis-like skin pathology. The ease and reproducibility of this model made it ideal for our study [17, 18]. Here, we show that peripheral skin inflammation can induce a ‘remote’ chemokine response in the brain that appears to be maintained independently of a response in the periphery. In addition, we show that this is associated with leukocyte infiltration into the brain parenchyma, a reduction in neural precursor cells in the dentate gyrus of the hippocampus and behavioural alterations reflected in a suppression of burrowing behaviour. These findings highlight the impact of peripheral immune stimulation on homeostatic CNS function and implicate chemokines and their receptors as potential therapeutic targets for chronic inflammatory disease associated neuropsychiatric co-morbidities.

## Methods

### Mice

Wild-type C57BL/6 female mice (6- to 8-week old, 16–20 g) were purchased from Harlan Laboratories. Mice were maintained in specific pathogen-free conditions in the Central Research Facility at the University of Glasgow. All experiments received ethical approval and were performed under the auspices of UK Home Office Licence.

### Models of inflammation

Several models of peripheral inflammation were used in this study. These included the topical application of a cream containing the TLR7/8 ligand IMQ (Aldara model), the topical application of a sterile inflammatory agent (TPA), the topical application of IMQ in the absence of active components in the Aldara vehicle (Topical IMQ) and the intraperitoneal administration of IMQ (soluble IMQ). The four models are described in detail below. Since three out of the four models were used to induce cutaneous inflammation, this study focused on female mice, as it has been shown that male mice respond differently to topically applied inflammatory agents [19].

(i) Aldara model of skin inflammation
  Mice were shaved on their dorsal skin 24 h prior to Aldara treatment. Eighty milligrams of 5 % imiquimod (Aldara™, MEDA Ab, Stockholm, Sweden) cream, or aqueous control cream, was applied to the shaved dorsal every 24 h for 1, 3 or 5 days. Mice were euthanised 24 h following the final application.
(ii) Topically applied IMQ model
  In accordance with manufacturer’s instructions, soluble IMQ (Source BioScience, Nottingham, UK) was reconstituted in PBS prior to being dissolved in aqueous control cream. Mice were shaved on their dorsal skin 24 h prior to treatment. Eighty milligrams of 5 % IMQ cream, or aqueous control cream, was applied every 24 h for 5 days. Mice were euthanised 24 h following the final application.
(iii) Soluble IMQ injection model
  Soluble IMQ was reconstituted in PBS to a concentration of 1 mg/ml. Mice were injected, intraperitoneally, with 100 μl IMQ (100 μg) or PBS every 24 h for 5 days. Mice were euthanised 24 h following the final application.
(iv) TPA model of inflammation
  Mice were shaved on their dorsal skin 24 h prior to treatment. One hundred fifty microlitres of 100 μM 12-O-tetradecanoylphorbol-13-acetate (TPA) (Sigma Aldrich, Missouri, USA), or an equal volume of acetone control (Sigma-Aldrich, Missouri, USA), was applied to the mice every 24 h for 1, 3 or 5 days. Mice were euthanised 24 h after the final application.

### Cardiac perfusion of animal tissues

Prior to tissue retrieval, all mice were extensively perfused as follows: the right atrium of the heart was cut to allow blood to drain into the chest cavity without compromising the circulation. Perfusions were performed by injecting with 20 ml of PBS, warmed to 37 °C, into the left ventricle of the beating heart using a 23G needle.

### RNA isolation from brain tissue and peripheral blood leukocytes

Brain tissue was ‘snap-frozen’ and stored at −80 °C until use. Under RNase-free conditions, brains were homogenised using the TissueLyser LT (Qiagen, Hilden, Germany). RNA was extracted from homogenised tissue using QIAzol® Lysis Reagent (Qiagen, Hilden, Germany), isolated RNA was further purified and genomic DNA was removed, using an RNeasy Mini Kit (Qiagen, Hilden, Germany). Red blood cells were lysed from blood samples using red blood cell lysis buffer (Miltenyi, Cologne, Germany). Cells were first passed through a QIAshredder (Qiagen, Hilden, Germany) before RNA was isolated from peripheral blood leukocytes (PBL) using an RNeasy Mini Kit.

### GeneChip microarray analysis

Microarray analyses were performed in the Glasgow Polyomics Facility at the University of Glasgow (www.polyomics.gla.ac.uk), as previously described [20]. Briefly, 1 μg of purified total RNA was amplified by in vitro transcription and converted to sense-strand complementary DNA (cDNA) using a WT Expression kit (Life Technologies, California, USA). Fragmented and labelled cDNA samples were hybridised to GeneChip Mouse Gene 1.0 ST Arrays (Affymetrix, California, USA). Procedures were carried out as described by the manufacturers. Microarray profiling data were deposited in the National Center for Biotechnology Information Gene Expression Omnibus database with the series entry identifier GSE72214 and can be accessed using the following link: http://www.ncbi.nlm.nih.gov/geo/query/acc.cgi?token=sbmpmemufzynhmj&acc=GSE72214.

Data generated using GeneSpring GX software were normalised using RMA 16. Normalised data were analysed using unpaired *t* tests to determine the significance of each gene in Aldara-treated mice compared to control mice. *p* values were adjusted for multiple comparisons using the Benjamini Hochberg multiple comparison test.

Gene ontology terms were assigned to differentially expressed genes using the Database for Annotation, Visualization and Integrated Discovery (DAVID) Bioinformatics Resources v6.7 (http://david.abcc.ncifcrf.gov/). Analysis was performed in accordance with two protocols outlined by Huang et al. [21, 22]. The significance of enrichment was determined using a modified Fisher’s exact test. A Benjamini-Hochberg multiple comparison test was used to correct for the rate of type I errors. Co-expression of a gene cluster was considered significant if it satisfied a *p* value cutoff of 0.05.

### QRT-PCR

Total RNA was reverse transcribed using Quantitect® Reverse Transcription kit (Qiagen, Hilden, Germany) with random primers. Quantitative real-time PCR (QRT-PCR) amplifications were performed in triplicate using PerfeCTa® SYBR® Green FastMix® (Quanta Biosystems, Maryland, USA). Primers were designed using Primer3 Input software (version 0.4.0) and generated by IDT Technologies. Primer sequences are listed in Additional file 1: Table S1. A 750-nM mix of forward and reverse primers was used per reaction. QPCR reactions were performed using a Prism® 7900HT Sequence Detection System (Life Technologies, California, USA) for 40 cycles in accordance with manufacturer’s guidelines. The absolute copy number was calculated from a standard curve and normalised to the housekeeping gene, TATA-binding protein (TBP), as previously described [23]. Fold-change values were calculated by comparing the normalised copy number of individual samples to the mean of the control samples.

### Luminex

Blood was collected by cardiac puncture. Plasma was isolated from whole blood by centrifugation. Plasma concentrations of soluble inflammatory mediators were determined using mouse multiplex cytokine Luminex panel kits (Life Technologies, California, USA) in accordance with manufacturer’s instructions.

### Generation of a single-cell suspension from brain tissue

Perfused brains were extracted from control and treated mice as described. Brains were digested for 45 min at 37°, 750 rpm in 10 ml digestion buffer (6 μg/ml Liberase TM (Roche), 5 U/ml DNaseI and 25 mM Hepes buffer diluted in HBSS (all Sigma Aldrich, Missouri, USA)). Following digestion, cell suspensions were passed through a 70-μm cell strainer before being washed twice with 2-mM EDTA in HBSS. Myelin removal was performed using myelin removal beads (Miltenyi Biotech, Cologne, Germany) as per the manufacturer’s instructions using an AutoMACS. Total cell number was determined using a haemocytometer.

### Flow cytometry

Cells were first incubated with 1-μl FcR block (Miltenyi) per sample and then stained at 4 °C using the antibodies listed in Additional file 2: Table S2. Samples were analysed using an LSR II or FACSAria I/III cytometer (BD Biosciences) and FlowJo software (Tree Star).

### Legendplex protein assay

Chemokine protein expression was measured using the Legendplex assay (BioLegend, California, USA) as per the manufacturer’s instructions. In brief, snap-frozen brain tissue was homogenised in N-PER™ Neuronal Protein Extraction Reagent (Thermo Scientific) at a ratio of 10 ml per 1 g of tissue for 20 min on ice. Samples were centrifuged at 10,000*g* for 10 min, and supernatant was collected. Legendplex beads were incubated with whole brain lysate for 2 h at 600 rpm on a plate shaker. Beads were conjugated with streptavidin-PE for 30 min and were washed twice prior to sample reading using BD LSRII flow cytometer. Samples were differentiated on the basis of bead size and APC fluorescence. Protein quantity was determined using SA-PE fluorescence calibrated to a standard curve. Limits of detection were 1.69 and 1.4 pg/ml for CCL2 and CXCL10, respectively.

### Histology

Brain and skin samples were fixed in 10 % neutral buffered formalin prior to processing and paraffin embedding. Processing was performed using the Shandon Citadel 1000 automated tissue processor (Thermo scientific).

(i) *Haematoxylin and eosin staining*
  Skin samples from treated mice were cut into 5-μm sections and stained with haematoxylin and eosin (H&E) to discern morphology.
(ii) *CD3 T cell staining*
  Brains extracted from Aldara-treated mice were formalin fixed and embedded in paraffin as described. CD3 staining was performed, using a rabbit anti-mouse CD3 antibody (Vector), by the Veterinary Diagnostic Service Facility at the University of Glasgow. Slides were cut from three sequential regions through the mid-sagittal brain, from four mice per group. Slides were blinded and whole sections were counted for CD3.
(iii) *Doublecortin (DCX) staining for neurogenesis*
  Seven micrometre sections were cut from formalin-fixed brains from mice treated with Aldara or control cream for 5 days. Sections were stained with polyclonal goat anti-mouse DCX (Santa Cruz Biotechnology, Texas, USA). Slides were blinded, and the mean DCX counts from three areas of the dentate gyrus of the hippocampus were obtained per section.

### Burrowing model of mouse behaviour

The assessment of burrowing behaviour was carried out as previously described [24]. Briefly, groups were acclimatised overnight prior to baseline tests being performed. Forty-eight hours later, mice were put on procedure and were treated with Aldara or control cream as described above. Burrowing tests were carried out on individually caged mice for a 2-h time period, 4 h after Aldara treatment, for three consecutive days.

### Statistical analysis

All data were analysed using the Prism 4 software (GraphPad, San Diego, CA, USA). Results are shown as mean ± standard deviation (SD) unless stated otherwise. Data were analysed using the unpaired two-tailed student’s *t* test if two groups were compared. If more than two groups were compared a one- or two-way ANOVA was performed using the Bonferroni post-test. The figure legends indicate which statistical test was used for each graph. A *p* value of ≤0.05 was considered as statistically significant.

## Results

### Aldara-treatment-induced psoriasis-like skin inflammation is associated with a remote transcriptional response in the brain

**Fig. 1:**
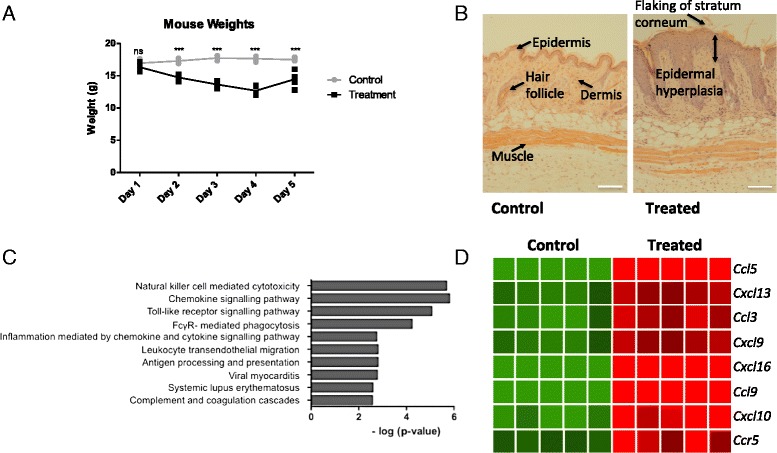
Aldara application, which causes a psoriasis-like skin inflammation, induces a remote transcriptional response in the brain. Mice were treated with 80 mg of Aldara cream or control cream every 24 h for five consecutive days. **a** Mouse weights were recorded after each treatment. Mice were euthanised 24 h after the final application, and **b** areas of treated skin were sectioned to 5 μm, stained with H&E and visualised at ×100 magnification using a light microscope. Epidermis [1], dermis [2], hair follicles [3], muscle [4], flaking of the stratum corneum [5] and epidermal hyperplasia [6] are shown. *Scale bar* = 100 μm. Microarray analysis was performed on perfused whole brains. **c** DAVID analysis was used to identify enriched biological pathways. **d** GeneSpring GX software was used to identify differentially expressed chemokine genes, which are presented on a heatmap and shown in order of fold-change induction in the brain. *n* = 5 mice per group. Significance was measured using two-way ANOVA with Bonferroni multiple comparison post-tests ***p ≤ 0.001

In this study, we performed a full genome microarray to identify genes that may be differentially expressed in the brain during the response to cutaneous Aldara treatment. Mice treated with Aldara lost a significant amount of body weight. This weight loss, shown in Fig. 1a, was apparent after just one application and was most pronounced between the third and fifth treatments. Aldara treatment also induced psoriasis-like skin inflammation in the mice, causing visible erythema and flakiness. The psoriatic symptoms were confirmed in H&E sections (Fig. 1b) taken 24 h after the fifth application of Aldara, which show epidermal and dermal hyperplasia, keratosis and marked skin thickening. To test the hypothesis that peripheral inflammation induces inflammatory changes in the brain, we comprehensively evaluated alterations in gene expression in the brain in response to cutaneous Aldara treatment using a full genome microarray approach. When analysed using GeneSpring GX software, over 200 transcripts were significantly upregulated in the brains of Aldara-treated mice compared with control mice when a stringent fold-change cutoff of 3 or above was applied. DAVID software was used to determine the biological associations of the differentially expressed genes by grouping them based on their function (Fig. 1c). This analysis highlighted immune, inflammatory and leukocyte-derived genes as being prominent within the differentially expressed functional gene groupings. Interestingly, amongst the top ten functional annotations, *chemokine signalling pathway*, *inflammation mediated by chemokine and cytokine signalling pathway* and *leukocyte transendothelial migration* were enriched. As they play a critical role in homeostatic and inflammatory responses, both in the periphery and the CNS, we focused on chemokines as a potential mechanism of communication between the periphery and the brain. Amongst the subset of genes that were differentially expressed between the two groups, seven chemokines and one chemokine receptor were upregulated in the brains of Aldara-treated mice, with a fold-change induction ranging between 3-fold and 25-fold. The relative expression levels of these eight genes, *Ccl3*, *Ccl5*, *Cxcl13*, *Cxcl9*, *Cxcl16*, *Ccl9*, *Cxcl10* and *Ccr5*, are displayed in the heatmap in Fig. 1d, where they are presented in order of their fold-change induction. Taken together, these data suggest a remote upregulation of chemokine expression in the brain in response to localised cutaneous inflammatory responses.

### Aldara treatment induces the upregulation of a subset of chemokines in the brain, independent of a measurable inflammatory profile in the circulation

To confirm the upregulated expression of chemokine transcripts in the brains of Aldara-treated mice, and to define the time course of induction, an independent experiment was performed and chemokine gene expression was analysed after one, three and five applications of Aldara using QRT-PCR. To determine whether these transcriptional changes arose in the brain independently of residual signals from contaminating blood cells left over from the perfusion, we also measured expression levels in the peripheral blood cells from the treated mice.

**Fig. 2:**
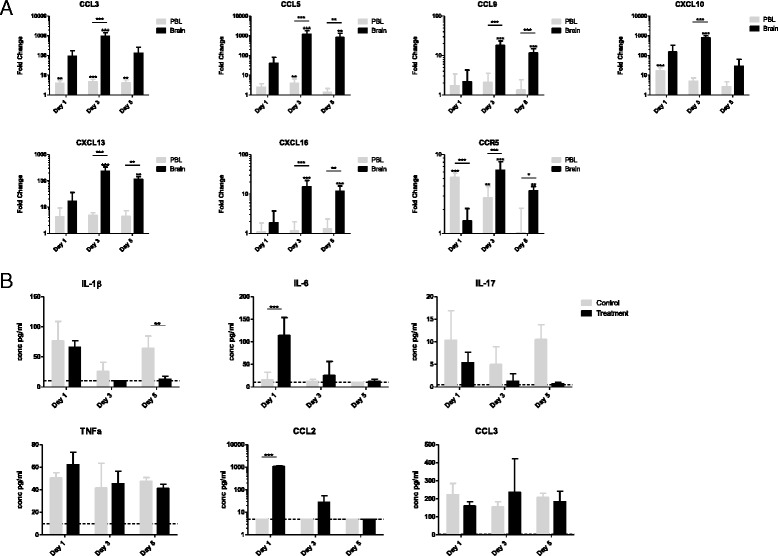
Aldara treatment induces a transcriptional brain response that is independent of a systemic peripheral response. Mice were treated with 80 mg Aldara cream or control cream every 24 h for one, three or five consecutive days. Mice were euthanised 24 h after the final application. Cardiac puncture was performed to retrieve PBLs, and perfused brains were extracted. RNA was isolated from both tissues, and **a** QRT-PCR analysis of the target chemokine genes was performed. **b** Luminex analysis was performed to determine the expression of classic inflammatory cytokines in the plasma. *n* = 4 mice per group. Significance was measured using two-way ANOVA with Bonferroni multiple comparison post-tests. Additional two-way ANOVA were used to compare the control and treated groups within each tissue (**a**). ***p ≤ 0.001 **p ≤ 0.01 *p ≤ 0.05

At day 1, 24 h after topical Aldara treatment, *Ccl3*, *Cxcl10* and *Ccr5* were significantly upregulated by PBL in the Aldara-treated group compared to those isolated from the control group (Fig. 2a, Additional file 3: Figure S1). At day 3, *Ccl3* and *Ccl5* remained significantly elevated by PBL from Aldara-treated mice; however, by day 5, *Ccl5* expression had returned to baseline. Only *CCL3* remained significantly elevated by this time point (Fig. 2a, Additional file 3: Figure S1). Thus, although there is chemokine induction in the periphery, this was largely restricted to the earlier time points.

In contrast to the mild chemokine response observed in PBL, topical Aldara treatment resulted in a potent and sustained chemokine induction in the brain. By day 3, all seven genes were significantly upregulated in the brains of Aldara-treated mice (Fig. 2a). Strikingly, the expression of *Ccl3*, *Ccl5* and *Cxcl10* was increased almost 1000-fold at this time point. With the exception of *Ccl3* and *Cxcl10*, the expression of all genes remained significantly elevated at day 5. This was particularly notable for *Ccl5*, the expression level of which was approximately 900-fold greater than the control. Importantly, when comparing the fold induction in the brain to that of the PBL, expression of all chemokine/chemokine receptor target genes was increased to a significantly greater level in the brain than PBL at day 3 of the Aldara model (Fig. 2a). Therefore, alterations to the transcriptional profile of the brain were not a consequence of residual peripheral blood contamination. Again, with the exception of *Ccl3* and *Cxcl10*, the fold-change induction remains significantly greater in the brain than the PBL at day 5. *Ccr5* was the only gene that shows a significantly greater fold-change induction in the PBL than in the brain. However, this was only observed on day 1 of the Aldara model. Although the microarray data demonstrated a significant induction of the gene encoding CXCL9, this appeared to be below the detection limit of the QRT-PCR assay and could not be validated in the PBL and the brain (data not shown). It was therefore excluded from further analysis. Together, these data confirm that topical Aldara treatment triggers a large increase in chemokine transcription in the brain. Importantly, the response in the brain differs both in magnitude and temporal patterning to that in the PBL, with chemokine transcription remaining elevated throughout the model.

To investigate whether the transcriptional response in the brain was being driven by the presence of circulating inflammatory cytokines, protein levels of IL-1β, IL-6, IL-17, TNF-α, CCL2 and CCL3 were assessed at different time points using luminex (Fig. 2b). IL-6 and CCL2 were the only proteins to be significantly increased in the treatment group compared with the control group, and this was only the case after the first treatment. None of the other inflammatory cytokines were significantly increased in the circulation at day 1, day 3 or day 5. Collectively, the results in Fig. 2 demonstrate that the brain response to Aldara treatment differs from the peripheral blood response and does not appear to be associated with the expression of archetypal inflammatory cytokines in the circulation.

### Topically applied soluble IMQ induces chemokine induction in the brain without causing overt skin inflammation

**Fig. 3:**
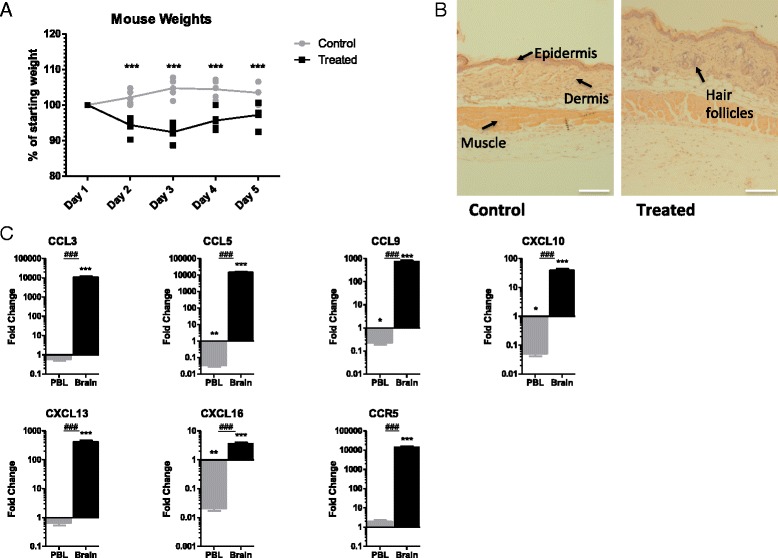
Topically applied imiquimod induces a transcriptional brain response but does not induce psoriasis-like skin inflammation. Mice were treated with 80 mg of control cream containing 5 % imiquimod or control cream alone every 24 h for five consecutive days. **a** Mouse weights were recorded after each treatment and are shown as percentage of initial weight, which was considered 100 %. Mice were euthanised 24 h after the final application, and **b** areas of treated skin were sectioned to 5 μm, stained with H&E and visualised at ×100 magnification using a light microscope. Epidermis [1], dermis [2], muscle [3] and hair follicles [4] are shown. *Scale bar* = 100 μm. PBL and perfused brains were collected, and **c** QRT-PCR analysis of the target chemokine genes was performed. *n* = 5 mice per group. Significance was measured using two-way ANOVA with Bonferroni multiple comparison post-tests. Individual unpaired student’s *t* tests were used to compare control vs treated within each tissue (**c**). *** (###) = *p* ≤ 0.001 ** (##) = *p* ≤ 0.01 *(#) = *p* ≤ 0.05

There are reports that, along with the active TLR agonist, imiquimod, Aldara cream contains another active component: isostearic acid. This component of the vehicle is thought to activate the inflammasome, induce keratinocyte cell death and stimulate the production of certain pro-inflammatory cytokines [25]. To further explore the mechanisms by which the brain-specific chemokine response is induced following Aldara treatment, and to discriminate between the activities of the isostearic acid and IMQ, soluble IMQ was dissolved in isostearic acid-free aqueous control cream and applied topically to the dorsal skin. As before, mice treated with soluble IMQ lost a significant amount of weight after the first two treatments (Fig. 3a). Their weights remained significantly lower than the control group throughout the model. In contrast to Aldara treatment, topical soluble IMQ treatment was not associated with significant skin inflammation. The skin sections, examined after five applications of IMQ cream, do not show any overt signs of epidermal hyperplasia, keratosis or erythema (Fig. 3b). These data suggest that this aspect of the response may be independent of TLR stimulation and reliant instead on the actions of isostearic acid. Nonetheless, all chemokine/chemokine receptor target genes were significantly elevated in the brain following topical IMQ treatment, independently of alterations in PBL (Fig. 3c). Of note, *Ccl3*, *Ccl5* and *Ccr5* presented with a fold-change induction upwards of 1000-fold. In this model, all genes encoding chemokine ligands were downregulated in the PBL of treated mice compared with control mice. With regard to all genes, the fold-change induction in the brain following treatment was significantly higher than the fold change in PBL. These results demonstrate that, although the different active components of Aldara may be responsible for driving different aspects of the response, the actions of TLR ligand IMQ alone are sufficient to induce the remote differential chemokine response in the brain.

### The differential chemokine response is dependent on topical application, as systemically induced IMQ does not induce a similar brain response

To determine whether the IMQ-induced response was reliant on topical application to the skin, intraperitoneal injections of IMQ were performed over the same period of time. In contrast to the previous models, this treatment did not induce any weight loss over the 5-day period and the weights of the treated mice increased gradually in line with the control group (Additional file 4: Figure S2A). In stark contrast to the potent chemokine response that was induced in the brain following topical Aldara and/or IMQ application, only *Ccl5* and *Ccr5* were significantly elevated in the brain (Additional file 4: Figure S2B) and this induction was modest (approximately threefold and 1.2-fold, respectively). Further, the induction of *Ccl5* and *Ccr5* expression in the brain did not differ in magnitude to that of the PBL. These findings clearly demonstrate that injecting IMQ systemically does not generate a similar response to the topical application of IMQ. Thus, the induction of chemokines in the brain is likely dependent, at least in part, on the initial skin response.

### Topical application and TLR ligation are essential for the induction of chemokines in the brain

Having shown that the effects of cutaneous Aldara cream treatment are mediated primarily through the actions of the TLR ligand IMQ, we compared the response to a model which generates a similar skin phenotype without acting through TLRs. TPA is a chemical irritant which, when applied to the dorsal skin, generates a similar skin profile of epidermal and dermal hyperplasia and erythema. However, this model does not act through TLRs and is thus considered ‘sterile’. In contrast to both the Aldara and topical IMQ models, topical TPA treatment did not induce weight loss in the treated mice (Additional file 5: Figure S3A). However, TPA did cause a similar skin inflammation to that observed following topical Aldara treatment (Fig. 1b, Additional file 5: Figure S3B). This was again characterised by skin thickening, epidermal and dermal hyperplasia, keratosis and erythema. Interestingly, none of the chemokine/chemokine receptor target genes were significantly altered in the brains or PBL of TPA-treated mice (Additional file 5: Figure S3C). Furthermore, in the plasma, only CCL2 was strongly and significantly differentially expressed, and this induction was restricted to day 1 (Additional file 5: Figure S3D). Circulating levels of all other inflammatory cytokines and chemokines were comparable between the treatment and control groups. Together, these data suggest that the brain response that occurs following topical Aldara treatment is dependent on skin-specific TLR ligation and is not simply a generic response to skin inflammation.

### Aldara treatment induces the infiltration of monocytes/macrophages into the brain

The most characterised role of chemokines is to coordinate leukocyte migration [26]. Therefore, we used flow cytometry to establish whether the transcriptional upregulation of chemokines in the brain following topical Aldara treatment was associated with an influx of leukocytes from the periphery. To exclude resident microglia that express intermediate levels of CD45, we only gated on live, single, CD45^hi^ cells (Additional file 6: Figure S4A). The results show a significant increase in the number of CD45^hi^ cells present in the brains at both day 3 and day 5 following Aldara treatment (Additional file 6: Figure S4B). Thus, topical Aldara treatment results in leukocyte recruitment to the brain.

**Fig. 4:**
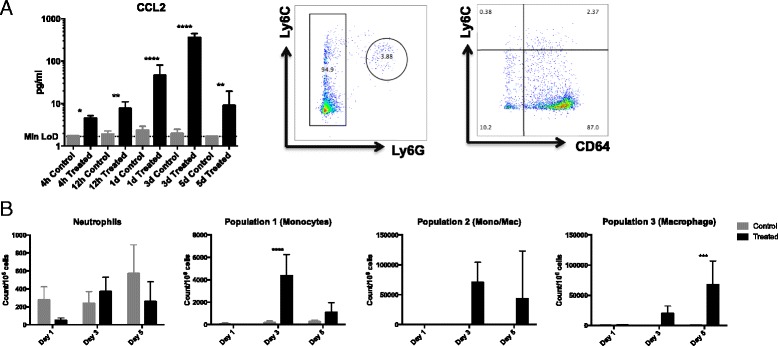
Aldara treatment induces the infiltration of monocytes, but not neutrophils, into the brain. Mice were treated with 80-mg Aldara cream or control cream every 24 h for one, three or five consecutive days. Mice were euthanised 24 h following the final application. **a** Protein expression of CCL2 in whole brain lysate was assessed in control and treated brains 4 h, 12 h, 1 day, 3 days and 5 days after treatment. **b** Perfused brains were homogenised, and a single cell suspension was generated as described. Cells were labelled with fluorescent antibodies. CD45^hi^CD11b^+^ cells were selected following dead cell exclusion and doublet exclusion, and representative gating strategy based on expression of Ly6C, Ly6G and CD64 is shown. **c** Counts per million cells were generated using total cell counts and are shown for neutrophils, monocytes, differentiating monocytes (population 2) and macrophages (population 3) at 1, 3 and 5 days following Aldara treatment. *n* = 4 mice per group. Significance was determined using one- (**a**) or two-way (**b**, **c**) ANOVA with Bonferroni multiple comparison post-tests. Data presented in **a** were log transformed prior to statistical analysis ****p < 0.0001 ***p ≤ 0.001 **p ≤ 0.01 *p ≤ 0.05

Monocytes are early responders to infection and have been shown to infiltrate the brain in peripheral inflammatory models and in response to viral infections that invade the central nervous system [44]. Interestingly, several of the chemokine transcripts that are elevated in the brain in response to topical Aldara treatment, specifically *Ccl3*, *Ccl5* and *Ccl9*, are known to recruit monocytes to sites of inflammation. In addition, protein analysis from an independent experiment clearly demonstrates that levels of CCL2, a classic monocyte chemoattractant, are also elevated in the brain following Aldara treatment (Fig. 4a). As shown in Additional file 6: Figure S4B, the treated brain appears to be devoid of infiltrating leukocytes at day 1. This prompted us to also analyse chemokine levels at two earlier time points, 4 and 12 h after treatment in order to capture the protein signal before and after leukocyte infiltration. Interestingly, CCL2 was significantly elevated in the brain from as early as 4 h following topical Aldara treatment and prior to leukocyte infiltration (Fig. 4a) These data suggest that CCL2 is likely to be produced by brain-resident cells, rather than the infiltrating leukocytes themselves.

Due to the enriched expression of monocyte chemoattractants in the brain throughout the Aldara model, we decided to use flow cytometry to determine whether the influx of leukocytes to the brain at days 3 and 5 were due to monocyte accumulation. Figure 4b shows the representative gating strategy used to identify neutrophils, monocytes and macrophages, from live, single, CD45^hi^CD11b^+^ cells. Consistent with there being no evidence of neutrophil chemoattractants being induced in the brain following Aldara treatment, there was no significant difference in the number of neutrophils between control and treated brains at any time point, with all counts being less than 600 per million cells (Fig. 4c). There was a substantial increase in monocyte numbers, defined as Ly6C^hi^Ly6G^−^CD64^−^ cells, in the brain at day 3 following Aldara treatment (Fig. 4c). Although it was not statistically significant, a notably high number of Ly6C^hi^CD64^+^ cells could also be detected in the brains of treated mice at this time point. By day 5, the number of monocytes and Ly6C^hi^CD64^+^ cells (population 2) appeared to reduce, whilst the number of Ly6C^low^CD64^+^ cells (population 3) significantly increased (Fig. 4c). This would suggest the infiltrating monocytes begin to differentiate into tissue macrophages in the brain between day 3 and day 5.

### Aldara treatment triggers the infiltration of CD4^+^ T cells, CD8^+^ T cells, NK cells and NK T cells into the brain

**Fig. 5:**
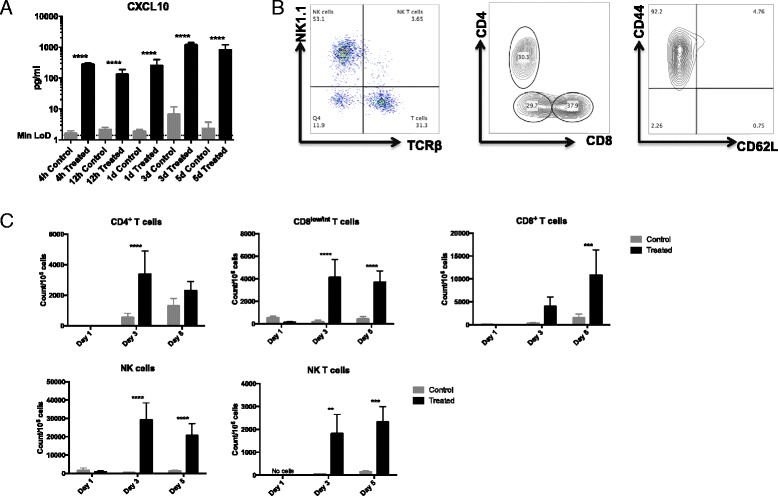
Aldara treatment induces the infiltration of a number of lymphocyte subsets into the brain. Mice were treated with 80-mg Aldara cream or control cream every 24 h for one, three or five consecutive days. Mice were euthanised 24 h following the final application. **a** Protein expression of CXCL10 in whole brain lysate was assessed in control and treated brains 4 h, 12 h, 1 day, 3 days and 5 days after treatment. **b** Perfused brains were homogenised, and a single cell suspension was generated as described. Cells were labelled with fluorescent antibodies. CD11b^−^ cells were selected following dead cell exclusion, and doublet exclusion and representative gating strategy based on expression of NK1.1, TCRb, CD4, CD8, CD44 and CD62L is shown. **c** Counts per million cells were generated using total cell counts and are shown for NK cells, NK T cells, CD8^lo^ T cells, CD8^+^ T cells and CD4^+^ T cells at 1, 3 and 5 days following Aldara treatment. *n* = 4 mice per group. Significance was determined using one- (**a**) or two-way (**b**, **c**) ANOVA with Bonferroni multiple comparison post-tests. Data shown in **a** were log transformed prior to statistical analysis ****p < 0.0001 ***p ≤ 0.001 **p ≤ 0.01

In addition to recruiting monocytes to sites of inflammation, CCL3 and CCL5 can also recruit populations of lymphocytes, as can CXCL10, CXCL13 and CXCL16. Thus, we next sought to characterise the full lymphocyte repertoire in the brain at the three different time points. To determine whether the transcription of lymphocyte chemoattractants in the brain resulted in elevated protein production, we performed protein analysis of a key T cell-recruiting chemokine, CXCL10. Again, in an attempt to address whether the source of the chemokine signature we report in this journal is brain-resident or from infiltrating cells, we also analysed CXCL10 expression at 4 and 12 h after treatment. CXCL10 protein expression was significantly induced in the treated brain as early as 4 h after cutaneous Aldara treatment when compared with control brain (Fig. 5a). CXCL10 remained significantly elevated throughout the model, with the highest expression level at day 3. Whilst we cannot comment on the source of the signal at day 3 and day 5, when there appears to be a mixed population of brain-resident cells and leukocyte infiltrate, we suggest it is unlikely that CXCL10 is being produced by infiltrating leukocytes at the earlier time points.

Figure 5b represents the gating strategy used to identify NK cells, NK T cells and CD4^+^ and CD8^+^ T cells, which were pre-gated as live, single, CD11b^−^ cells. Surprisingly, although *Cxcl13* expression was strongly induced in the brain following treatment, there was no evidence of a CD19^+^ B cell infiltrate at any of the time points (data not shown). We saw little evidence of any lymphocyte infiltration after the first treatment; however, a significant increase in lymphocyte number was clear at day 3 and day 5 post-treatment (Fig. 5c). TCRβ^+^ T cells could be divided into the conventional CD4+ and CD8+ subsets. However, there was an additional population of T cells that, rather than expressing the classic ‘double negative’ T cell profile (i.e. CD4^−^CD8^−^), expressed low levels of CD8 (CD8^lo^ T cells) (Fig. 5b). These cells expressed similar levels of both TCRβ and CD3 (data not shown). The results show that NK cells, NK T cells, CD8^lo^ T cells and CD4^+^ T cells were all significantly increased in the brains of Aldara-treated mice at day 3 (Fig. 5c). NK cells were found to be the most abundant subset, with an average cell count just below 30,000 cells/10^6^ cells in the treated brains.

By day 5, numbers of NK cells, NK T cells and CD8^lo^ T cells remained significantly elevated in the brains of treated mice (Fig. 5c). CD8^+^ T cell numbers did not become significantly increased in the brain until day 5 of the Aldara model, at which point the numbers of CD4 T cells had returned to normal. Consistent with previous studies showing that only activated T cells can enter the brain, the vast majority of infiltrating T cells displayed an effector/memory phenotype (Fig. 5b).

**Fig. 6:**
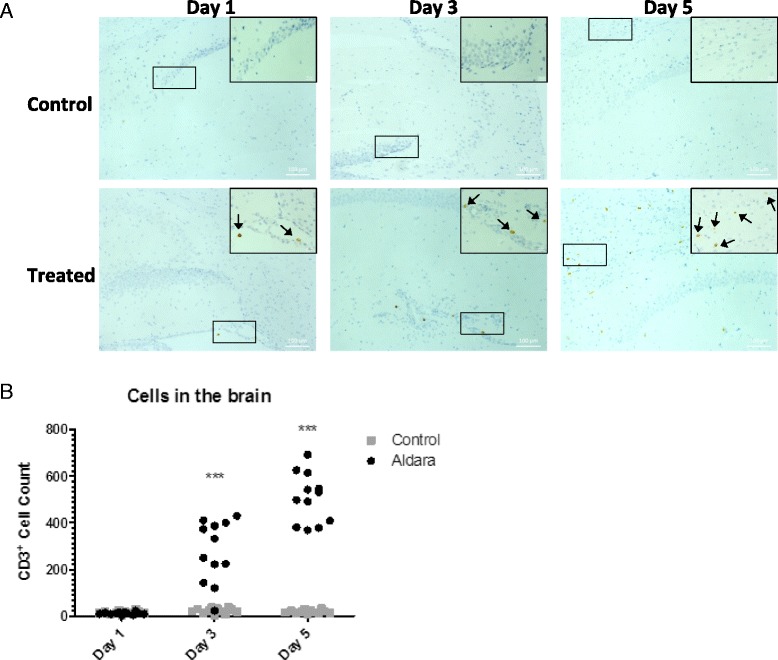
Aldara treatment induces the infiltration of CD3^+^ cells into the brain. Mice were treated with 80-mg Aldara cream or control cream every 24 h for one, three or five consecutive days. Mice were euthanised 24 h following the final application. Perfused brains were fixed in formalin and embedded in paraffin before being sectioned to 7 μm. **a** Three sections from each brain were stained for CD3. Representative sections of the hippocampus and cerebellum are shown at ×400 magnification or ×100 magnification (□). *Arrows* indicate CD3^+^ cells. **b** Whole brain CD3^+^ infiltrates were counted blind. *n* = 4 mice per group. Significance was determined using two-way ANOVA with Bonferroni multiple comparison post-tests ***p ≤ 0.001

Together, these data characterise the significant lymphoid infiltrate into the brain following cutaneous Aldara treatment and implicate brain-resident cells as an initial source of the T cell chemoattractant CXCL10. Surprisingly, histological examination of CD3 expression in the brain revealed that, rather than being localised to perivascular cuffs, CD3+ cells were scattered throughout the brain parenchyma (Fig. 6a). There was no anatomical specificity to the infiltrating cells as they were present throughout the parenchyma, as well as around specific brain regions including the cerebellum, hippocampus and circumventricular organs (CVOs). Consistent with the flow cytometry data, there were elevated numbers of CD3+ cells at day 3 and day 5 following Aldara treatment (Fig. 6b).

### Cutaneous Aldara treatment causes a reduction in neurogenesis and a suppression in burrowing activity

**Fig. 7:**
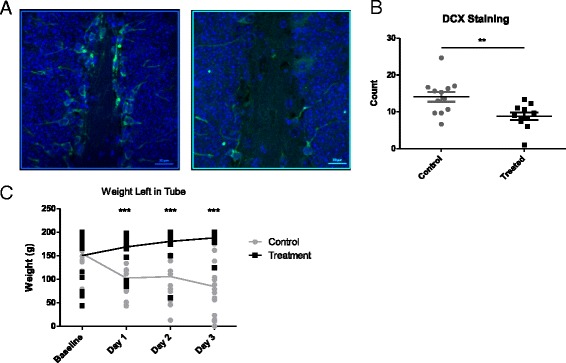
Aldara treatment causes a reduction of adult neurogenesis and a suppression of burrowing activity. Mice were treated with 80-mg Aldara cream or control cream every 24 h for one, three or five consecutive days and were euthanised 24 h after the final application. **a** Brains were isolated from mice after five applications of Aldara and were formalin fixed and paraffin embedded. Seven micrometre sections were cut and immunostained for DCX and were visualised using a fluorescent microscope at ×400 magnification **b** Blind counts of three areas of the dentate gyrus were performed for each section to determine the quantity of new neurons. **c** Burrowing activity was determined 4 h after one, two and three treatments of Aldara cream. Data are presented as the weight of material left in the burrowing tube after a 2-h test period. *n* = 5 mice per group for neurogenesis (mean of three areas from 11/12 sections per group), *n* = 10 mice per group for burrowing assessment. Significance was determined using unpaired student’s *t* test (**b**) or two-way ANOVA with Bonferroni multiple comparison post-tests (**c**). ***p ≤ 0.001 **p ≤ 0.01

In order to determine the consequences of the Aldara-induced transcriptional response in the brain, a molecular output, neurogenesis, and a functional output, burrowing behaviour, were assessed. Neuroinflammation has been shown to have a negative effect on hippocampal neurogenesis during neurodegenerative diseases and following the administration of LPS and inflammatory cytokines [27–30]. In addition, there is a link between neurogenesis and behaviour and some have reported that impaired neurogenesis is associated with hippocampal cognitive impairments, learning deficits and depressive-like symptoms [31–33]. The extent to which adult neurogenesis was affected in this study was evaluated by counting the number of doublecortin (DCX)-positive neurons in the dentate gyrus of the hippocampus. DCX is a microtubule-associated protein expressed by neuronal precursor cells and immature neurons in embryonic and adult cortical structures. As there is almost exclusive expression of DCX in developing neurons, it has been used increasingly as a marker for neurogenesis. Figure 7a shows representative staining of the dentate gyrus in both control and Aldara-treated mice 24 h following the fifth application. The amount of green stain suggested that Aldara treatment caused a reduction in the number of DCX-positive neurons. This was quantified by performing blinded cell counts, which confirmed that Aldara treatment caused a significant reduction in the quantity of neurogenesis (Fig. 7b). Our results are in agreement with previously published data suggesting that inflammation can have a negative impact on neurogenesis.

To examine possible behavioural correlates of increased brain chemokine expression and T cell accumulation, we next undertook behavioural analyses. As a behavioural read-out, a basic burrowing model, in which mice burrow for food pellets, was employed. The read-out is the weight of pellets remaining in the burrowing tubes, as opposed to that burrowed out of the tubes, after defined periods of time. Burrowing is an innately rewarding activity in rodents; thus, this model indicates the level of well-being in mice based on the amount of burrowing activity that they participate in. As such, this model has been used in various studies to identify a behavioural output following inflammatory stimuli [34, 35]. Mice were acclimatised to the burrowing tubes prior to treatment, and baseline tests were performed. Whilst the baseline tests showed a wide range of burrowing activity, the Aldara-treated and control groups began to separate after only one treatment. Figure 7c shows the weight of pellets left in the tube following a 2-h testing period after each application. The control mice had less weight left in the tubes than treated mice, meaning that burrowing activity was higher; whereas Aldara-treated mice burrowed less with each application. Unfortunately, this test could not be continued over the full duration of the treatment period as it has been reported that a decline in performance may occur due to over-exposure to the procedure [36]. These custom-made burrowing tubes were designed in such a way that they did not interfere with the area of treated dorsal skin; and it was observed throughout the model that the treated mice displayed a normal range of physical ability, for example climbing on the bars of the cage. Therefore, this significantly impaired burrowing activity indicates that cutaneous Aldara treatment has a negative effect on the well-being of the mice.

## Discussion

This study demonstrates that peripherally induced, tissue-specific inflammation can ‘remotely’ alter the transcriptional profile of the brain, independently of a similar response in the periphery. Topical Aldara treatment, which induces a psoriasis-like skin inflammation, causes the upregulation of a number of genes in the brain, including seven inflammatory chemokines and one chemokine receptor, a response that was not mimicked by PBL. This response peaks following the third application but persists beyond the fifth application suggesting a prolonged expression in the brain. That this response was also seen with topically applied reconstituted IMQ, but not with a sterile model of skin inflammation, implicates peripheral TLR ligation as an important mechanism in the induction of this response. In addition, the lack of a brain response following an I.P. injection of reconstituted IMQ suggests that the localised skin response is also an important factor in driving the brain response.

The use of reconstituted IMQ yielded an interesting result in that a significant brain response was observed in the absence of an overt skin inflammation, which is important when considering the mechanisms of communication between the periphery and the brain. Although the lack of cutaneous inflammation is reportedly due to the omission of isostearic acid [25], it is difficult to hypothesise how the effects of peripheral inflammation are being transmitted to the brain without the generation of a local inflammatory response or circulating inflammatory cytokines. It is also notable that neither induction of a ‘sterile’ cutaneous inflammatory response nor intraperitoneal administration of IMQ resulted in the generation of a brain response in our study. One possibility that might unite these disparate observations is that the communication between the inflamed skin and the brain is not driven by inflammatory agents but by direct ligation of TLRs on sensory nerves within the skin which then transmit a response to the brain inducing transcriptional changes associated with ‘sickness behaviour’. A number of studies have shown TLR expression on peripheral nerves [37–39], and a further series of experiments is planned by our lab to specifically address this issue. An alternative hypothesis is that tissue-resident myeloid cells, or other leukocytes exposed to the inflamed environment in the skin, could transmit the signal to the brain directly, by migrating through the circulation to the brain. Alternatively, they could transmit inflammatory signals indirectly, by causing damage to peripheral nerves that is then sensed by the brain. Whilst we do not feel this provides a mechanistic explanation for our observations, due to the fact that TPA treatment induces a potent inflammatory response in the skin and is not associated with chemokine upregulation in the brain, further studies would be required to investigate this potential route of immune-to-brain communication.

Strikingly, in addition to triggering the expression of chemokines in the brain, topical Aldara treatment was associated with a sizable recruitment of several leukocyte populations to the brain, including CD4^+^ T cells, CD8^+^ T cells, NK cells, NK T cells and monocytes. On day 3, the monocyte populations in the brain were Ly6C^+^CD64^-^ and Ly6C^+^CD64^+^, whereas by day 5, they were predominantly Ly6C^+^CD64^+^ monocytes and Ly6C^-^CD64^+^ macrophages. This is likely indicative of monocyte to macrophage differentiation. Although we cannot directly link enhanced chemokine production to leukocyte recruitment, the chemokine profile in the brain was highly specific to the cell populations recruited.

Several groups have shown that chemokine induction in the brain can induce leukocyte infiltration, both under neuroinflammatory conditions [16, 40–43], in response to peripheral inflammation [44] and during CNS viral infection [16, 45]. The brain specific chemokine profile that is triggered following topical Aldara treatment bears a notable resemblance to the array of chemokines that are induced in the brain following encephalitic virus infection [16]. Indeed, the leukocyte populations that infiltrate the brain following Aldara treatment: monocytes, effector memory T cells, NK cells and NKT cells, are populations that play a prominent role during viral infection and are often crucial for pathogen clearance. Furthermore, as would be expected during viral encephalitis, IMQ, the active component of Aldara, stimulates TLR7 to mimic a viral response. Thus, in response to TLR7 ligation in the periphery, the brain may specifically produce monocyte and lymphocyte chemoattractants to recruit the appropriate populations required to protect the brain against a potential viral infection.

The infiltration of immune cells into the brain following a remote immune challenge is both surprising and interesting. The generation of a cellular brain response following peripheral inflammation could indicate the presence of a protective surveillance mechanism initiated when a potential CNS insult is anticipated to ‘prime’ the brain. However, further studies would need to be performed before we could begin to unpick the role of these infiltrating cells, and their fate beyond the time points examined.

A key question regarding this response relates to the temporal relationship between chemokine induction in the brain and leukocyte recruitment, as infiltrating leukocytes may either have responded to chemokines made in the brain prior to their recruitment, or may in fact have contributed to the expression of the detected chemokines. However, at least two key inflammatory chemokines, CXCL10 and CCL2, are upregulated in the brain at the protein level by 4 h respectively following topical Aldara treatment, whereas immune cells are not recruited into the brain until 72 h post-treatment. This leads us to propose that chemokine production within the brain parenchyma precedes, and is likely to be at least partially responsible for, the subsequent recruitment of immune cells.

In addition to immune cell infiltration, chemokines are also implicated in a number of neuromodulatory processes, including neurotransmission, development and neurogenesis [11, 46–48], indicating that changes in chemokine expression could disrupt essential homeostatic events in the CNS [49]. In this study, we have been able to link peripheral cutaneous inflammation with two relevant functional outputs: an impairment in adult neurogenesis at the dentate gyrus and a reduction in burrowing activity of mice, a behaviour that has been shown to be a sensitive representation of neuropsychiatric imbalance in a number of inflammatory CNS disease models [24, 50]. It is worth noting that whilst food pellets were a convenient material to be used in large quantities, it is assumed that this model is not appetite-dependent and mice were not ‘food rationed’ at any time. Nevertheless, it remains possible that an additional contribution to the lack of burrowing seen in the inflamed mice may come from a generalised lack of interest in food as a consequence of an impaired sense of well-being. Of course, to fully characterise the behavioural deficits in response to Aldara treatment, and to associate these with neuropsychiatric conditions, more sophisticated, impairment-specific behavioural tests should be performed. Nonetheless, our data suggest that inflammation generated at distant sites has the potential to negatively influence homeostatic brain function.

## Conclusions

In summary, this study shows that peripheral, tissue-specific skin inflammation can remotely induce both transcriptional and functional responses in the brain, and it implicates inflammatory chemokines as potential mediators of the brain response to peripheral inflammation. This study has provided a comprehensive characterisation of the peripherally triggered, infiltrating leukocytes in the brain, which have the potential to exert a profound effect on CNS homeostasis. Our findings highlight the sensitivity of the CNS to peripheral changes and offer a correlative insight into how chronic inflammatory diseases are commonly associated with the onset of neuropsychiatric co-morbidities. With further investigation, it is hoped that the intricate mechanisms can be unravelled and key mediators identified, thus allowing for better treatment strategies for these co-morbid disorders.

## Additional files

Additional file 1: Table S1. Fluorescent antibodies used for lymphoid* and myeloid** cell flow cytometry analysis. (PNG 70 kb) Additional file 2: Table S2. Primer sequences used for QRT-PCR analysis. (PNG 47 kb) Additional file 3: Figure S1. Transcriptional PBL response of Aldara-treated mice. Mice were treated with 80 mg Aldara cream or control cream every 24 h for 1, 3 or 5 consecutive days. Mice were euthanised 24 h after the final application. Cardiac puncture was performed to retrieve PBLs and RNA was isolated from the tissue. (A) QRT-PCR analysis of the target chemokine genes was performed. *n* = 4 mice per group. Significance was measured using two-way ANOVA with Bonferroni multiple comparison post-tests ****p* ≤ 0.001 ***p* ≤ 0.01 **p* ≤ 0.05. (PNG 32 kb) Additional file 4: Figure S2. Imiquimod injected intraperitoneally does not induce a transcriptional brain response. Mice were injected intraperitoneally with 100 μl (100 mg/ml) of Imiquimod or PBS every 24 h for 5 consecutive days. (A) Mouse weights were recorded after each treatment. Mice were euthanised 24 h after the final application, PBL and perfused brains were collected and (B) QRT-PCR analysis of the target chemokine genes was performed. *n* = 5 mice per group. Significance was measured using two-way ANOVA with Bonferroni multiple comparison post-tests. Individual unpaired student’s t tests were used to compare control vs treated within each tissue. ***p* ≤ 0.01 **p* ≤ 0.05. (PNG 40 kb) Additional file 5: Figure S3. TPA treatment causes psoriasis-like skin inflammation but does not induce a transcriptional response in the brain. Mice were treated with 100 μg of TPA or an equal volume of acetone every 24 h for 1, 3 or 5 consecutive days. (A) Mouse weights were recorded after each treatment. Mice were euthanised 24 h after the final application and (B) areas of treated skin were sectioned to 5 μm, stained with H&E and visualised at ×100 magnification using a light microscope. Epidermis [1], dermis [2], muscle [3] and epidermal hyperplasia [4] are shown. Scale bar = 100 μm. (C) QRT-PCR analysis of the target chemokine genes was performed for PBL and brains at 1, 3 and 5 treatments (D) Luminex analysis was performed to assess the expression of classic inflammatory cytokines in the plasma at 1,3 and 5 treatments. *n* = 4 mice per group. Significance was measured using two-way ANOVA with Bonferroni multiple comparison post-tests. In addition, two-way ANOVA were used to compare control vs treated within each tissue at the different time points (C). (PNG 131 kb) Additional file 6: Figure S4. Identification of CD45^hi^ leukocytes in the brain following Aldara treatment. Mice were treated with 80 mg Aldara cream or control cream every 24h for 1, 3 or 5 consecutive days. Mice were euthanised 24h after the final application. Perfused brains were homogenised to generate a single cell suspension and were analysed using flow cytometry. (A) leukocytes were identified based on CD45^hi^ expression and (B) numbers of infiltrating cells in the brains of control and treated mice are shown as counts/10^6^ cells. *n* = 4 mice per group. Significance was measured using two-way ANOVA with Bonferroni multiple comparison post-tests ****p* ≤ 0.001 ***p* ≤ 0.01. (PNG 42 kb)

## Abbreviations

CNS: central nervous system
CVO: circumventricular organ
DAVID: database for annotation, visualisation and integrated discovery
IMQ: imiquimod
MDD: major depressive disorder
PBL: peripheral blood leukocyte
pDC: plasmacytoid dendritic cell
TBP: TATA-binding protein
TLR: Toll-like receptor
TPA: 12-O-tetradecanoylphorbol-13-acetate
