# Supplementary figures and images for: TLR7-mediated skin inflammation remotely triggers chemokine expression and leukocyte accumulation in the brain
Source: J Neuroinflammation. 2016 May 9;13:102. doi: 10.1186/s12974-016-0562-2 (PMC4862138; doi:10.1186/s12974-016-0562-2)

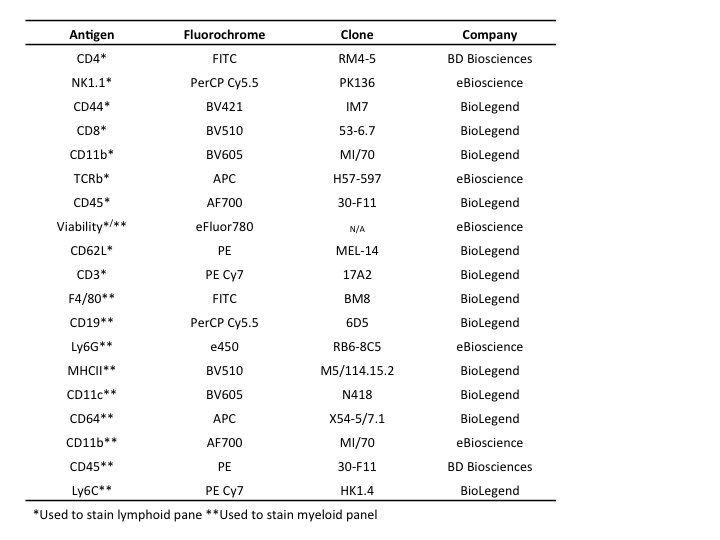

Supplement: Additional file 1: Table S1. — Fluorescent antibodies used for lymphoid* and myeloid** cell flow cytometry analysis. (PNG 70 kb) [file 12974_2016_562_MOESM2_ESM.png]

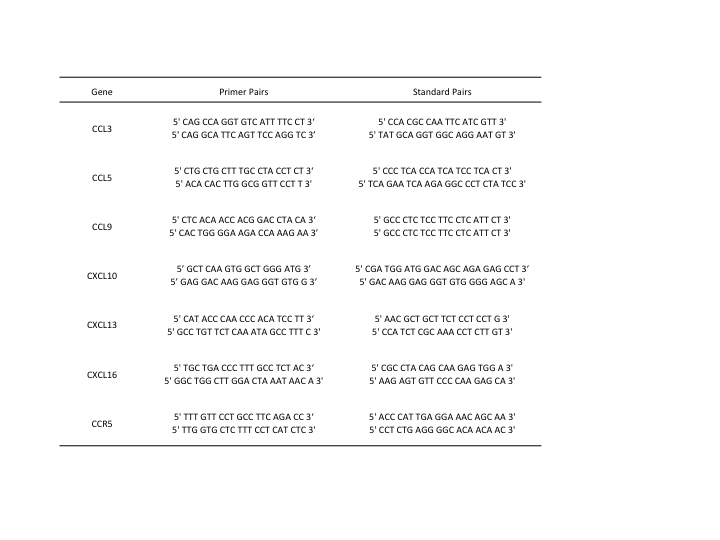

Supplement: Additional file 2: Table S2. — Primer sequences used for QRT-PCR analysis. (PNG 47 kb) [file 12974_2016_562_MOESM1_ESM.png]

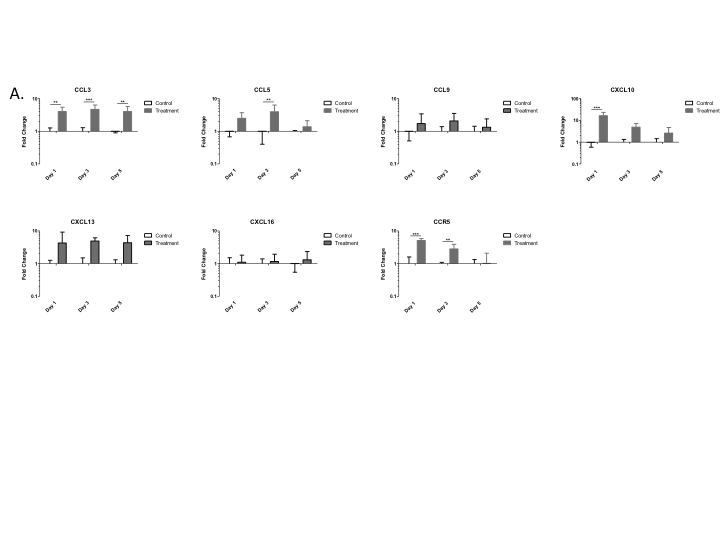

Supplement: Additional file 3: Figure S1. — Transcriptional PBL response of Aldara-treated mice. Mice were treated with 80 mg Aldara cream or control cream every 24 h for 1, 3 or 5 consecutive days. Mice were euthanised 24 h after the final application. Cardiac puncture was performed to retrieve PBLs and RNA was isolated from the tissue. (A) QRT-PCR analysis of the target chemokine genes was performed. n = 4 mice per group. Significance was measured using two-way ANOVA with Bonferroni multiple comparison post-tests ***p ≤ 0.001 **p ≤ 0.01 *p ≤ 0.05. (PNG 32 kb) [file 12974_2016_562_MOESM3_ESM.png]

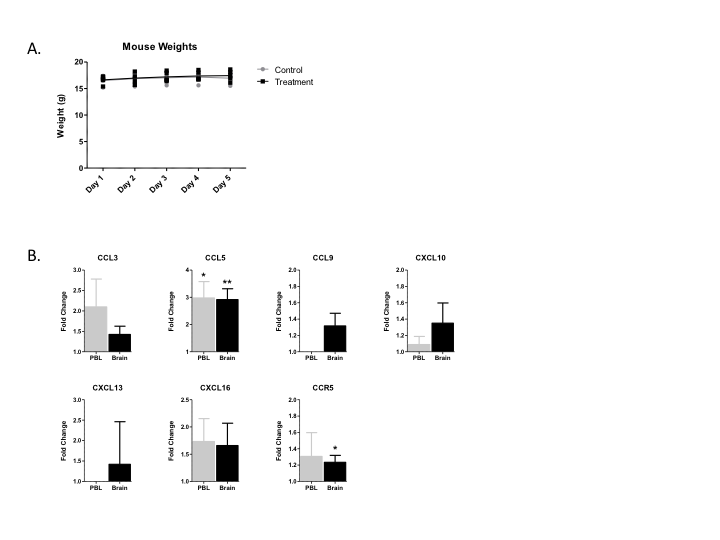

Supplement: Additional file 4: Figure S2. — Imiquimod injected intraperitoneally does not induce a transcriptional brain response. Mice were injected intraperitoneally with 100 μl (100 mg/ml) of Imiquimod or PBS every 24 h for 5 consecutive days. (A) Mouse weights were recorded after each treatment. Mice were euthanised 24 h after the final application, PBL and perfused brains were collected and (B) QRT-PCR analysis of the target chemokine genes was performed. n = 5 mice per group. Significance was measured using two-way ANOVA with Bonferroni multiple comparison post-tests. Individual unpaired student’s t tests were used to compare control vs treated within each tissue. **p ≤ 0.01 *p ≤ 0.05. (PNG 40 kb) [file 12974_2016_562_MOESM4_ESM.png]

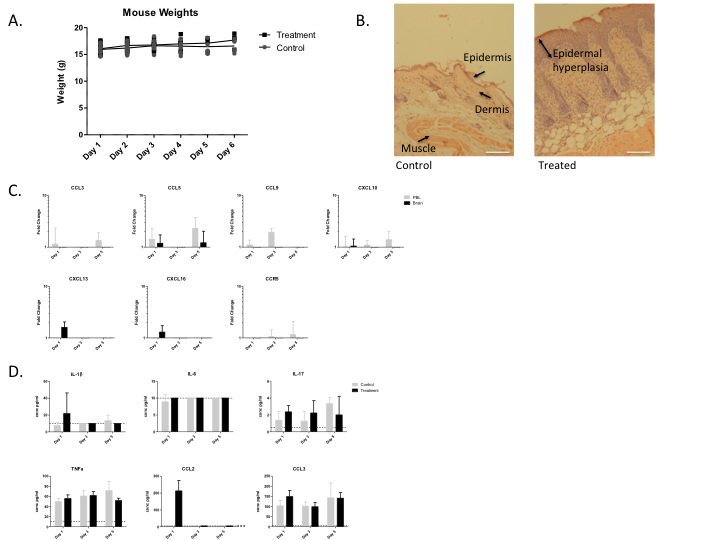

Supplement: Additional file 5: Figure S3. — TPA treatment causes psoriasis-like skin inflammation but does not induce a transcriptional response in the brain. Mice were treated with 100 μg of TPA or an equal volume of acetone every 24 h for 1, 3 or 5 consecutive days. (A) Mouse weights were recorded after each treatment. Mice were euthanised 24 h after the final application and (B) areas of treated skin were sectioned to 5 μm, stained with H&E and visualised at ×100 magnification using a light microscope. Epidermis [1], dermis [2], muscle [3] and epidermal hyperplasia [4] are shown. Scale bar = 100 μm. (C) QRT-PCR analysis of the target chemokine genes was performed for PBL and brains at 1, 3 and 5 treatments (D) Luminex analysis was performed to assess the expression of classic inflammatory cytokines in the plasma at 1,3 and 5 treatments. n = 4 mice per group. Significance was measured using two-way ANOVA with Bonferroni multiple comparison post-tests. In addition, two-way ANOVA were used to compare control vs treated within each tissue at the different time points (C). (PNG 131 kb) [file 12974_2016_562_MOESM5_ESM.png]

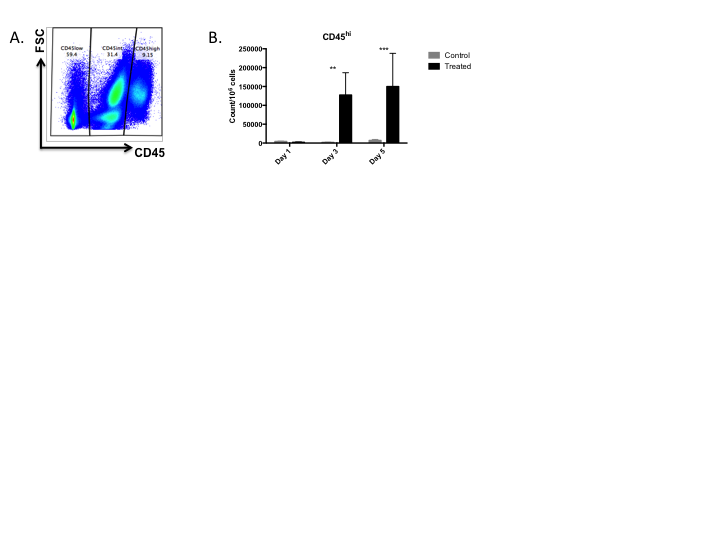

Supplement: Additional file 6: Figure S4. — Identification of CD45hi leukocytes in the brain following Aldara treatment. Mice were treated with 80 mg Aldara cream or control cream every 24h for 1, 3 or 5 consecutive days. Mice were euthanised 24h after the final application. Perfused brains were homogenised to generate a single cell suspension and were analysed using flow cytometry. (A) leukocytes were identified based on CD45hi expression and (B) numbers of infiltrating cells in the brains of control and treated mice are shown as counts/106 cells. n = 4 mice per group. Significance was measured using two-way ANOVA with Bonferroni multiple comparison post-tests ***p ≤ 0.001 **p ≤ 0.01. (PNG 42 kb) [file 12974_2016_562_MOESM6_ESM.png]
